# Monocytes Do Not Transdifferentiate into Proper Osteoblasts

**DOI:** 10.1100/2012/384936

**Published:** 2012-04-30

**Authors:** Andreas Schmitt, Sabrina Ehnert, Lilianna Schyschka, Peter Buschner, Andreas Kühnl, Stefan Döbele, Sebastian Siebenlist, Martin Lucke, Ulrich Stöckle, Andreas K. Nussler

**Affiliations:** ^1^Department of Traumatology, MRI, Technical University Munich, 81675 Munich, Germany; ^2^Department of Sports Orthopedics, “Klinikum rechts der Isar”, Technical University Munich, MRI, Ismaningerstrasse 22, 81675 Munich, Germany; ^3^Eberhard-Karls University, BG Trauma Clinic, 72076 Tübingen, Germany; ^4^Department of Vascular Surgery, MRI, Technical University Munich, 81675 Munich, Germany

## Abstract

Recent publications suggested that monocytes might be an attractive cell type to transdifferentiate into various cellular phenotypes. Aim was, therefore, to evaluate the potential of blood monocytes to transdifferentiate into osteoblasts. Monocytes isolated from peripheral blood were subjected to two previously published treatments to obtain unique, multipotent cell fractions, named programmable cells of monocytic origin (PCMOs) and monocyte-derived mesenchymal progenitor cells (MOMPs). Subsequently, MOMPs and PCMOs were treated with osteogenic differentiation medium (including either vitamin D or dexamethasone) for 14 days. Regarding a variety of surface markers, no differences between MOMPs, PCMOs, and primary monocytes could be detected. The treatment with osteogenic medium neither resulted in loss of hematopoietic markers nor in adoption of mesenchymal phenotype in all cell types. No significant effect was observed regarding the expression of osteogenic transcription factors, bone-related genes, or production of mineralized matrix. Osteogenic medium resulted in activation of monocytes and appearance of osteoclasts. In conclusion, none of the investigated monocyte cell types showed any transdifferentiation characteristics under the tested circumstances. Based on our data, we rather see an activation and maturation of monocytes towards macrophages and osteoclasts.

## 1. Introduction

Full recovery of large bone defects occurring after trauma or tumor is an unsolved problem in traumatology and orthopedic surgery as such defects can often only be treated insufficiently with conventional implants. As a consequence, patients frequently lose their mobility, which results in a loss of autonomy.

Recently, the concept of tissue engineering for replacing lost bone has been arousing great interest. In contrast to conventional implants, the engineered tissue construct will be integrated into the patient's tissue and replaced by newly formed bone, allowing entire recovery.

Beside the construction of a scaffold mimicking architecture and mechanical properties of the lost bone, the selection of cells seeded on this scaffold is critical. Ideally, cells are harvested from the patient himself to allow autologous therapy and avoid immunological reactions or transmission of infectious diseases. Primary bone cells are difficult to isolate in sufficient quantities. Hence, interest has mainly been focused on stem cells as the source for bone tissue engineering in the last decade. Multipotent adult stem cells have been identified in a number of tissues of the adult organism. They are responsible for maintaining the integrity of the tissue they reside in. Mesenchymal stem cells in particular have been proven to have a good potential to differentiate into osteoblasts [1]. They can be isolated from the patient's bone marrow or fat tissue. Nonetheless, harvesting mesenchymal stem cells requires invasive procedures that can cause serious morbidity [2]. Thus, opening alternative stem cell sources, which are accessible by less invasive procedures would progress the whole field of regenerative medicine.

In this context, reports about the inherent potential of peripheral blood monocytes to transdifferentiate into various cell types other than phagocytes have been increasing interest. They seem to inhere potential to transdifferentiate into cells of all three germ layers, namely, endothelial-, osteoblast-, chondrocyte-, myoblast-, hepatocyte-, epithelial-, neuronal-, keratinocyte-, smooth muscle-, pancreatic island- and adipocyte-like cells [3–8]. Thus, the differentiation potential of these cells even seems to exceed that of most adult stem cells. Additionally, autologous monocytes can be easily isolated by taking a blood sample. This offers great advantages compared to adult stem cells as source for autologous therapy. 

Two different methods have mainly been described to obtain a pluripotent cell fraction from peripheral blood monocytes. The first is the selection of a multipotent subset of the CD14 positive monocytes—termed “monocyte-derived mesenchymal progenitor cells” (MOMPs)—by cultivating them in presence of fibronectin and CD14 negative blood cells [9]. The alternative approach is a special dedifferentiation procedure, employing M-CSF, IL-3, and beta-mercaptoethanol, leading to “programmable cells of monocytic origin” (PCMOs) [7]. Both cell types have been shown to possess the potential for mesenchymal differentiation. MOMPs were shown to differentiate into osteoblast-like cells, producing mineralized matrix and expressing bone markers, such as alkaline phosphatase, osteocalcin, and bone sialoprotein [9]. PCMOs were successfully transdifferentiated into cells showing a chondrogenic phenotype. If PCMOs were treated with BMP2, they started expressing collagen type 1, which may be a hint for inherent potential for osteogenic transdifferentiation [10].

Concluding, peripheral blood monocytes seem to be a promising source for bone tissue engineering. The aim of the present study was to evaluate their potential use for bone tissue engineering.

## 2. Materials and Methods

### 2.1. Peptides, Antibodies, and Chemicals

Human recombinant M-CSF, IL-3, and RANKL were acquired from Peprotech (Peprotech, UK), human recombinant Fibronectin and Collagenase II were obtained from Biochrom (Biochrom, Germany); Mouse-anti-Human CD68 (DAKO, Denmark), FITC labeled Mouse-anti-Human CD14, CD45 (Biozol, Germany), APC-labeled Mouse-anti-Human CD90 (BioLegend, Netherlands), and PE-labeled mouse-anti-human CD105 (southern biotech, USA) antibodies were used; cell culture medium and supplements were provided by PAA (PAA, Germany), all other chemicals were obtained from Sigma (Sigma-Aldrich, Germany).

### 2.2. Ethical Statement

All used cell types were isolated from patients undergoing total hip replacement in the Department of Traumatology, MRI, Technical University Munich. The study was approved by the ethics committee of the faculty of medicine of the Technical University of Munich (http://www.ek.med.tum.de/, Project Number 2413, TU Munich, Germany). Patients gave their written consent before surgery.

### 2.3. Isolation and Culture of Peripheral Blood Monocytes

Peripheral blood mononuclear cells were isolated by density gradient centrifugation with help of LSM 1077 (PAA, Germany) from whole blood. Mononuclear cells (100,000 cells/cm^2^) were seeded on uncoated culture flasks and cultured in presence of RPMI 1640 medium (10% autologous serum, 2 mM L-glutamine, 100 U/mL penicillin, 100 *μ*g/mL streptomycin). After 2 h of culture, nonadherent cells were carefully removed by repetitive washing with PBS.

Adherent cells were further cultivated in uncoated cell culture flasks in RPMI 1640 culture medium supplemented with 10% autologous serum, 2 mM L-glutamine, 100 U/mL penicillin, and 100 *μ*g/mL streptomycin, without addition of cytokines.

### 2.4. Selection and Culture of MOMPs

MOMPs were selected according to the protocol published by Kuwana et al. [9]. Briefly, mononuclear cells (100,000 cells/cm^2^) were seeded in fibronectin-coated (10 *μ*g/mL) culture flasks and cultured in presence of DMEM low glucose medium with 10% autologous serum, 2 mM L-glutamine, 100 U/mL penicillin, and 100 *μ*g/mL streptomycin without addition of cytokines. Cells were cultured for six days with regular change of medium under avoidance of removal of the nonadherent blood cells.

### 2.5. Dedifferentiation of Monocytes into PCMOs and Culture of PCMOs

PCMOs were dedifferentiated from monocytes according to the method published by Ruhnke et al. [7]. This was modified in use of autologous serum instead of human AB-serum [3]. Isolated mononuclear blood cells (100,000 cells/cm^2^) were seeded in uncoated culture flasks and cultured in presence of RPMI 1640 medium (10% autologous serum, 2 mM L-glutamine, 100 U/mL penicillin, 100 *μ*g/mL streptomycin) for two hours. Non-adherent cells were removed by gentle washing with PBS. Adherent cells were cultivated in dedifferentiation medium (RPMI 1640 medium, 10% autologous serum, 2 mM L-glutamine, 100 U/mL penicillin, 100 *μ*g/mL streptomycin, 5 ng/mL recombinant human M-CSF, 0.4 ng/mL recombinant human IL-3 and 140 *μ*M beta-mercaptoethanol) for 6 days. Dedifferentiation medium was replaced every third day. 

### 2.6. Isolation of Primary Human Osteoblasts, Mesenchymal Stem Cells (MSCs), and Nonadherent Bone Marrow Cells (naBMCs)

As control, primary osteoblasts, MSCs, and naBMCs were harvested.

For osteoblast isolation, femur heads were processed. Small pieces of cancellous bone were washed in PBS and subsequently digested with an equal volume of digestion buffer (PBS, 0.07% Collagenase II) for 1 hour at 37°C. The treated bone fragments were placed in osteoblast culture medium (MEM/Ham's F12, 10% FCS, 2 mM L-glutamine, 100 U/mL penicillin, 100 *μ*g/mL streptomycin, 50 *μ*M L-ascorbate-2-phosphate, 50 *μ*M beta-glycerol-phosphate) in cell culture flasks. After 7 days, the cells began to grow out. After reaching confluence, the cells were transferred to new cell culture flasks and expanded.

Isolation of MSCs and naBMCs required bone marrow which was washed out of cancellous bone with PBS. Mononuclear cells were separated using density gradient centrifugation with help of LSM 1077. Cells were cultured in DMEM high-glucose medium (10% FCS, 2 mM L-glutamine, 100 U/mL penicillin, 100 *μ*g/mL streptomycin) in uncoated cell culture dishes. After 48 hours, nonadherent cells were removed by washing with PBS. The remaining mesenchymal stem cells were further cultured and expanded, while the nonadherent cell fraction (naBMCs) was collected as control.

### 2.7. Osteogenic Differentiation Procedure

Osteogenic differentiation of monocyte-derived cells (MOMPs and PCMOs) and mesenchymal stem cells (MSCs) was induced by cultivation in osteogenic medium (culture medium supplemented with 50 *μ*M L-ascorbate-2-phosphate, 10 mM beta glycerophosphate, and either 100 nM dexamethasone or 5 *μ*M vitamin D) for 14 days.

For osteogenic stimulation, osteoblasts were cultivated in osteogenic stimulation medium (DMEM/HAM's-F12 1 : 1, 5% FCS, 1% Pen/Strep, 10 mM beta-glycerophosphate, 200 *μ*M L-ascorbate-2-phosphate, 25 mM HEPES, 1.6 mM calciumchloride, 100 nM dexamethasone) for 14 days. Osteogenic medium was changed every third day.

### 2.8. Maturation of Monocytes to Macrophages and Osteoclasts

Mononuclear cells were seeded (100,000 cells/cm^2^) in uncoated cell culture flasks in RPMI1640 medium supplemented with 10% serum, 2 mM L-glutamine, 100 U/mL penicillin, and 100 *μ*g/mL streptomycin. After 24 hours nonadherent cells were removed by careful washing with PBS. Maturation into macrophages was induced by supplementation of culture medium with 10 ng/mL M-CSF for 7 days [11]. Osteoclasts were obtained by cultivation of monocytes in presence of 20 ng/mL RANKL (from day 1 to 6) and 25 ng/mL M-CSF (from day 6 to 20) [12].

### 2.9. Alkaline Phosphatase (AP) Activity Assay

After washing with PBS, cells were incubated with substrate buffer (0.2% 4-nitrophenyl-phosphate disodium salt hexahydrate, 50 mM glycine, 1 mM MgCl_2_, 100 mM TRIS, pH 10.5) for 1 h. Formation of 4-nitrophenol (pNP) was determined photometrically at 405 nm. Quantification was performed using a calibration of pNP. The enzyme activity was either normalized to cell viability measured by Alamar blue assay according to the manufacturer's protocol (Biozol, Germany), or to relative protein levels determined by Sulforhodamine B (SRB) staining [13].

### 2.10. Alizarin Red Staining

After washing twice with PBS, cells were fixed in 99% ethanol for one hour at −20°C. Subsequently, the matrix was stained by addition of Alizarin red (0.5%; pH 4.0) for 10 min. Unbound Alizarin red was removed by extensive washing with H_2_O. Bound Alizarin red was solved in 10% cetylpyridiumchloride. Alizarin red was detected photometrically at 562 nm and quantified by a calibration of Alizarin red.

### 2.11. TRAP Staining

After washing twice with PBS, cells were fixed with fixation buffer (3.1% formaldehyde, 0.2% Triton X-100 in PBS) for 5 min at room temperature. Subsequently, cells were covered with staining buffer (0.01% Naphtol AS-MX Phosphate, 0.06% Fast Red Violet LB Salt, 1% N-N Dimethylformamid, 40 mM Na-Acetat, 10 mM Na_2_-Tartrat, pH 5) for 10 min at 37°C.

### 2.12. TGF-*β*, TNF-*α*, and RANKL Measurement

For measuring of cytokine concentrations, PCMOs were incubated with serum-free culture medium (RPMI 1640, 2 mM L-glutamine) for 48 h at 37°C. TGF-beta levels were measured by TGF-beta-reporter cells (MFB-F11) [14]. MFB-F11 cells were cultured for 48 h with cell culture supernatants. Resulting SEAP activity in the culture supernatant was measured according to the manufacturer's instructions with the Great EscAPe SEAP fluorescent detection kit (Clonetech, France). TNF-alpha and RANKL levels were measured by ELISA according to the manufacturer's protocol (Peprotech, UK).

The detected signals were normalized to relative protein levels determined by SRB assay.

### 2.13. Conventional RT-PCR

Total cellular RNA was isolated with Trifast according to the manufacturer's protocol (Peqlab, Germany). First-strand cDNA was synthesized from 1 *μ*g RNA according to the manufacturer's instructions using the Transcriptor High Fidelity cDNA synthesis kit (Roche, Germany). Primer sequences and the corresponding annealing temperatures are summarized in Table 1. Products resolved by gel electrophoresis in a 1.5% (w/v) agarose gel were visualized with ethidiumbromide. Number of amplification cycles was adjusted for each primer to terminate reaction in the exponential phase of the PCR. Intensity of bands was quantified employing ImageJ software (NIH, USA) and normalized to GAPDH.

### 2.14. Immunostaining

Cultured cells were fixed with 4% formalin for 10 min, followed by washing in dH_2_O. After this preparation, cells were dried and stored up to 4 weeks for immunostaining. Immunostaining was performed at room temperature. First, cells were watered in TRIS buffer for 15 min, followed by quenching endogenous peroxidase activity with 0.3% H_2_O_2_ solution for 30 min. After extensive washing with TRIS buffer, immunostaining for CD68 was performed using the DAKO REAL Detection System Peroxydase/DAB+ Rabbit/Mouse-Kit (DAKO, Denmark). The primary CD68-Antibody was used in a dilution of 1 : 2000. Counterstaining with haematoxylin was performed additionally.

### 2.15. Flow Cytometry

For FACS analysis, cells were washed with PBS to remove nonadherent cells and harvested mechanically. After fixation with 4% formaline for 10 min, cells were incubated with antibodies against CD14, CD45, CD90, or CD105 for 40 min at ambient temperature. Corresponding isotype controls were used according to standard protocols. Measurement was performed in a FACSCanto II (BD, Germany). Data were evaluated employing FlowJo software (Tree Star, USA).

### 2.16. Statistics

Results are shown as column graphs showing the mean ± standard error of the mean (SEM). Datasets were compared by one-way analysis of variance followed by Bonferoni's multiple comparison test (GraphPad Prism Software, El Camino Real, USA). *P* < 0.05 was taken as minimum level of significance.

## 3. Results

### 3.1. MOMPs and PCMOs Show a Comparable Phenotype

**Figure 1:**
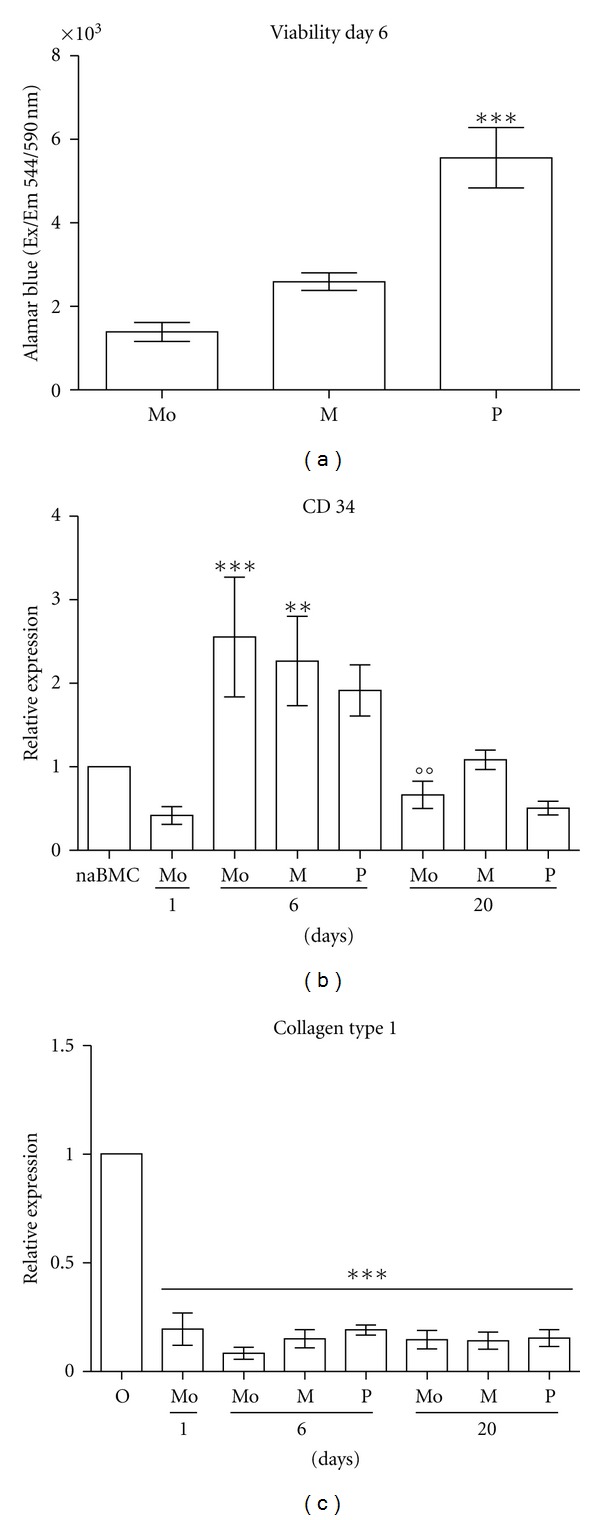
Treatments to obtain PCMOs and MOMPs influence proliferation and expression of monocytes. (a) Human mononuclear cells (*N* = 4, *n* = 3) were cultivated without specific treatment (Mo), treated for 6 days to obtain MOMPs (M) or PCMOs (P). Viability was measured by Alamar blue assay on day 6. P shows a significantly higher viability than Mo (***P < 0.001). (b) Relative expression levels of CD34 measured by RT-PCR normalized to GAPDH and subsequently to expression of CD34 in nonadherent bone marrow cells (naBMCs) as internal control (*N* = 3, *n* = 3). Mo and M show a significantly higher expression of CD34 on day 6 than Mo on day 1 (**P < 0.01; ***P < 0.001). After osteogenic differentiation, CD34 levels in Mo decreased significantly in comparison to Mo on day 6 (°°*P* < 0.05). (c) Relative expression levels of collagen type 1 measured by RT-PCR normalized to GAPDH and subsequently to expression of collagen type 1 in primary osteoblasts (O) as internal control (*N* = 3, *n* = 3). Mo, M and P show expression of low levels of collagen type 1 on all days (***P < 0.001). Monocytes (Mo) day 1: freshly isolated monocytes; Mo, MOMP (M), and PCMO (P) day 6: cells after treatment; Mo, M, P day 20: cells after differentiation with vitamin D containing medium.

Isolation of peripheral blood monocytes by gradient centrifugation and subsequent selection by adhesion resulted in approximately 60% CD14 and over 95% CD45 positive cells in FACS analysis (data not shown). Both treatments to obtain MOMPs and PCMOs from peripheral blood monocytes have a remarkable impact on the proliferation capacity of adherent cells. After 6 days of culture, PCMOs show significantly higher cell viability in comparison to untreated monocytes and MOMPs (Figure 1(a)).

Under both culture conditions to obtain MOMPs and PCMOs, as well as in the untreated control cells, two different cell morphologies were observed: one spindle-shaped, elongated form and a set of flattened, round cells. Here, the treatment to obtain PCMOs resulted in a population mainly consisting of the flattened, round phenotype after 6 days of culture, whereas MOMPs and untreated monocytes appeared as a mixed population with a considerable part of cells showing a spindle-shaped appearance.

On day 6 after isolation, MOMPs, PCMOs and untreated control monocytes were investigated for expression of hematopoietic markers CD14, CD34, CD45, CD68, and mesenchymal markers CD90, CD105, and collagen type 1.

**Figure 2:**
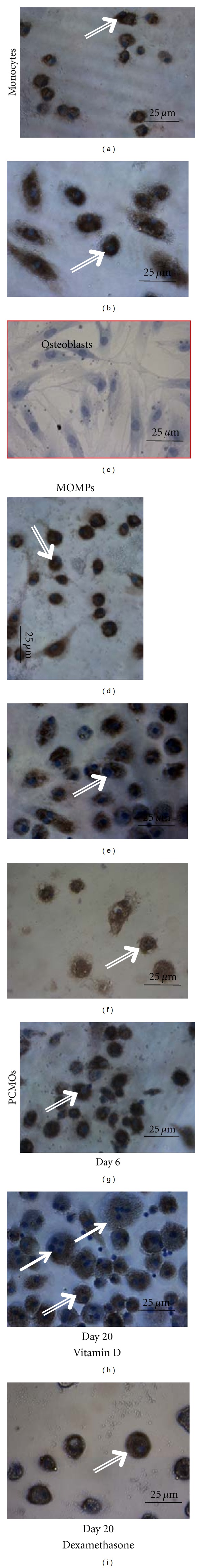
Differentiated monocytes (Mo), MOMPs (M), and PCMOs (P) express CD68. Representative picture of immunohistochemistry against CD68 in Mo, M, and P (*N* = 3, *n* = 3). After treatment to obtain MOMPs and PCMOs (“day 6;” first column) all cell types are positive for CD68. 20 days after the differentiation—either with vitamin D (column 2) or dexamethasone (column 3)—cells also expressed CD68, while primary osteoblasts proved to be negative for CD68. Magnification 200x. Following differentiation with vitamin D, high numbers of giant cells (marked with →) appear. Macrophage-like cells are marked with *⇒*.

Interestingly, the testing of the mentioned markers revealed that MOMPs and PCMOs share a similar phenotype with untreated control monocytes cultivated for the same time period. On day 6 all cell types express the markers CD14, CD45, CD90, and CD105 detected by flow cytometry (Table 2). In addition, all groups expressed similar levels of collagen type 1 and the hematopoietic stem cell marker CD34 (Figure 1). Immunostaining of MOMPs, PCMOs, and control monocytes showed that all of these were positive for the monocyte marker CD 68 (Figure 2).

### 3.2. MOMPs and PCMOs Show Weak Response to Differentiation Treatment Regarding Osteogenic Markers

To investigate the osteogenic potential of MOMPs and PCMOs, confluent cultures of both cell types were treated with osteogenic standard differentiation medium containing dexamethasone. We recognized a strong apoptotic effect of the dexamethasone-containing medium on all cultures, resulting in a decrease of cell number by more than 60% during 14 days and complete cell death after 28 days of treatment. Substitution of dexamethasone with vitamin D avoided these negative effects on cell viability completely (data not shown). Consequently, we used vitamin-D-containing medium for the following differentiation experiments.

To exclude beneficial effects of dexamethasone towards vitamin D regarding osteogenic differentiation, all differentiation experiments described below were performed with dexamethasone-containing differentiation medium in parallel. Here, dexamethasone showed no advantage towards vitamin-D-containing medium in any differentiation experiment (data not shown).

Treatment of MOMPs, PCMOs, and control monocytes, with differentiation media, resulted in the appearance of multinuclear giant cells. These represented a third phenotype, besides the persistent spindle-shaped and the elongated cells. Giant cells were found more frequently in cell populations treated with vitamin D than in those treated with dexamethasone-containing differentiation medium. They appeared in larger number in PCMO rather than in MOMP populations and in control monocytes and their number strongly varied among different donors. In some donors, they represented the major cell type after the differentiation procedure (Figure 2; giant cells marked with →).

**Figure 3:**
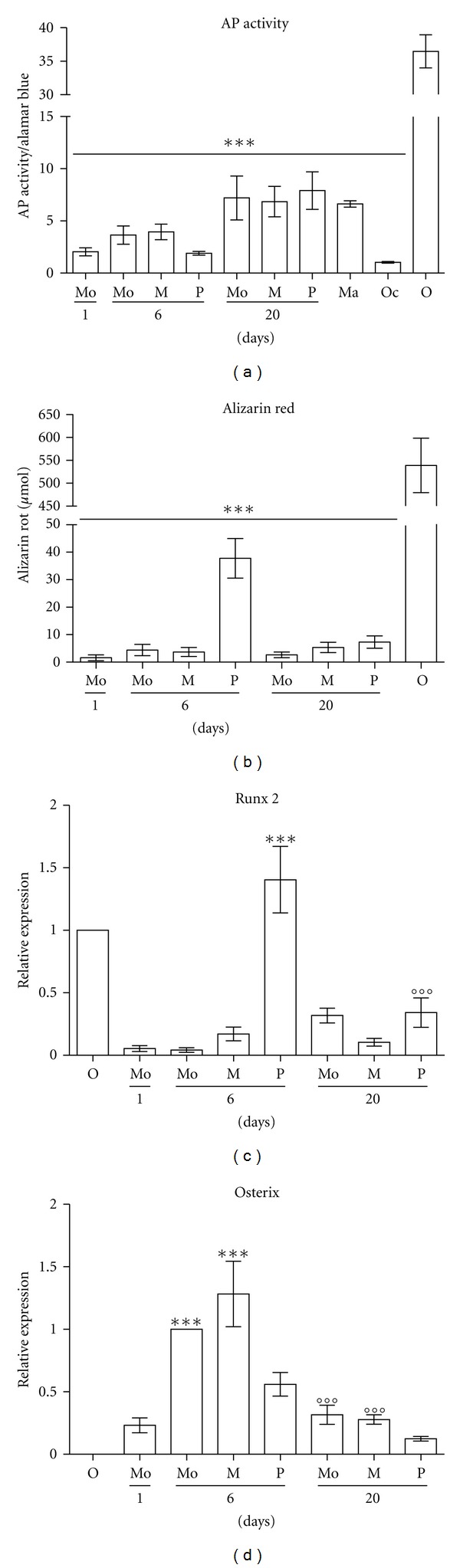
Weak response of monocyte-derived cells to differentiation. (a) Alkaline phosphatase (AP) activity [*μ*mol/min] of monocyte-derived cells in comparison to primary osteoblasts, macrophages, and osteoclasts (*N* = 4, *n* = 3). AP activity [*μ*M/min] normalized to cell amount determined by Alamar blue. (b) Alizarin red measurement [*μ*mol] in monocyte-derived cells in comparison to primary osteoblasts (*N* = 4, *n* = 3). (c) Relative expression levels of Runx2 measured by RT-PCR normalized to GAPDH and subsequently to expression in primary osteoblasts as internal control (*N* = 3, *n* = 3). Runx2 expression significantly increases after treatment to obtain PCMOs (***P < 0.001). After the differentiation procedure, Runx2 expression in PCMOs decreases again (°°°*P* < 0.001) (d) relative expression levels of Osterix measured by RT-PCR normalized to GAPDH and subsequently to expression in monocytes after 6 days in culture as internal control (*N* = 3, *n* = 3). Osterix expression significantly increases after treatment to obtain MOMPs and after 6 days culture of control monocytes (***P < 0.001). After the differentiation procedure, osterix expression in both cell types decreases again (°°°*P* < 0.001). Monocytes (Mo) day 1: freshly isolated monocytes; Mo, MOMP (M), and PCMO (P) day 6: cells after treatment; Mo, M, P day 20: cells after differentiation with vitamin-D-containing medium. Macrophages (Ma), osteoclasts (Oc). (***P < 0.001).

For investigation of alkaline phosphatase (AP), activity and production of mineralized matrix functional tests were performed. These are the most widely used markers to confirm osteogenic differentiation. Here, the differentiation procedure resulted in an increase in AP activity in both MOMPs and PCMOs, as well as in control monocytes. The achieved AP activity is low if compared to that of primary human osteoblasts (Figure 3(a)). In comparison to primary osteoblasts, MOMPs, PCMOs, and control monocytes produce minimal amounts of mineralized matrix after differentiation procedure (Figure 3(b)).

After observing a moderate effect of differentiation treatment on AP activity, we investigated whether or not differentiation treatment changes the expression of the bone-related transcription factors Runx2 and osterix by RT-PCR. As freshly isolated monocytes did not express Runx2, differentiation treatment of MOMPs, PCMOs, and control monocytes resulted in low Runx2 expression. However, the highest Runx2 expression, even reaching expression levels of primary osteoblasts, was observed in PCMOs without additional osteogenic differentiation treatment (Figure 3(c)). Expression levels of osterix decreased in MOMPs, PCMOs, and control monocytes after osteogenic differentiation treatment to levels observed in freshly isolated monocytes (Figure 3(d)).

**Figure 4:**
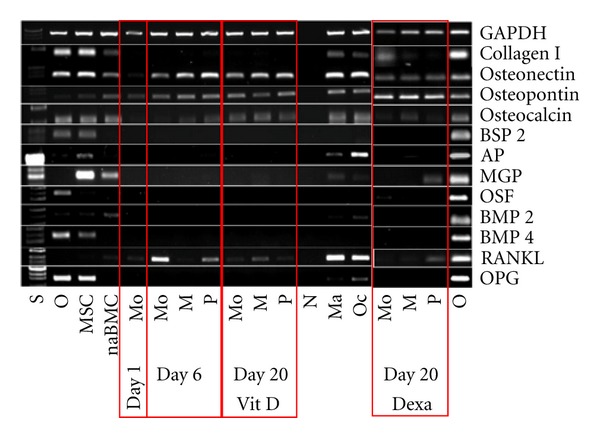
Differentiation treatment did not change bone marker expression. Expression pattern on important osteoblast marker gens detected by RT-PCR. Freshly isolated monocytes (Mo); M and P after treatment to obtain MOMPs and PCMOs and untreated control cells (Mo, M, P “day 6”), after differentiation with medium containing vitamin D (Mo, M, P “day 20 Vit. D”), or with medium containing dexamethasone (Mo, M, P “day 20 Dexa”); controls: macrophages (Ma), osteoclasts (Oc), primary osteoblasts (O), mesenchymal stem cells (MSC), nonadherent bone marrow cells (naBMC), and negative control (N). (*N* = 3, *n* = 1).

The effect of the differentiation procedure on osteogenic marker gene expression was investigated by RT-PCR. As expected from the expression profile of the two key regulators of osteogenesis (Runx2 and Osterix), the differentiation treatment had no substantial effect on expression levels of investigated osteogenic marker genes. These findings were similar in MOMPs and PCMOs treated with differentiation medium. Noteworthy, the bone markers osteonectin, osteopontin, and osteocalcin are already expressed at similar levels before differentiation treatment in MOMPs, PCMOs, freshly isolated monocytes, and untreated cultured monocytes (Figure 4).

### 3.3. Hematopoietic Cell Markers Persist during Dedifferentiation and Differentiation Procedure

As full transdifferentiation of monocytes into osteoblasts would also include losing hematopoietic markers, we were investigating if the differentiation treatment shows an impact on the expression of surface markers. Here, the osteogenic differentiation did not result in the loss of CD14 and CD45 expression, whereas markers of nonmaturated hematopoietic cells such as CD34 and CD90 decrease during the differentiation procedure (Figure 1(b), Table 2). Immunohistochemistry revealed that differentiated MOMPs, PCMOs, and control monocytes are positive for the monocyte marker CD68. The persistence of CD68 and the cells typical morphology identifies these rather as macrophages than as differentiated osteoblast-like cells (Figure 2; macrophage like cells marked with *⇒*).

**Figure 5:**
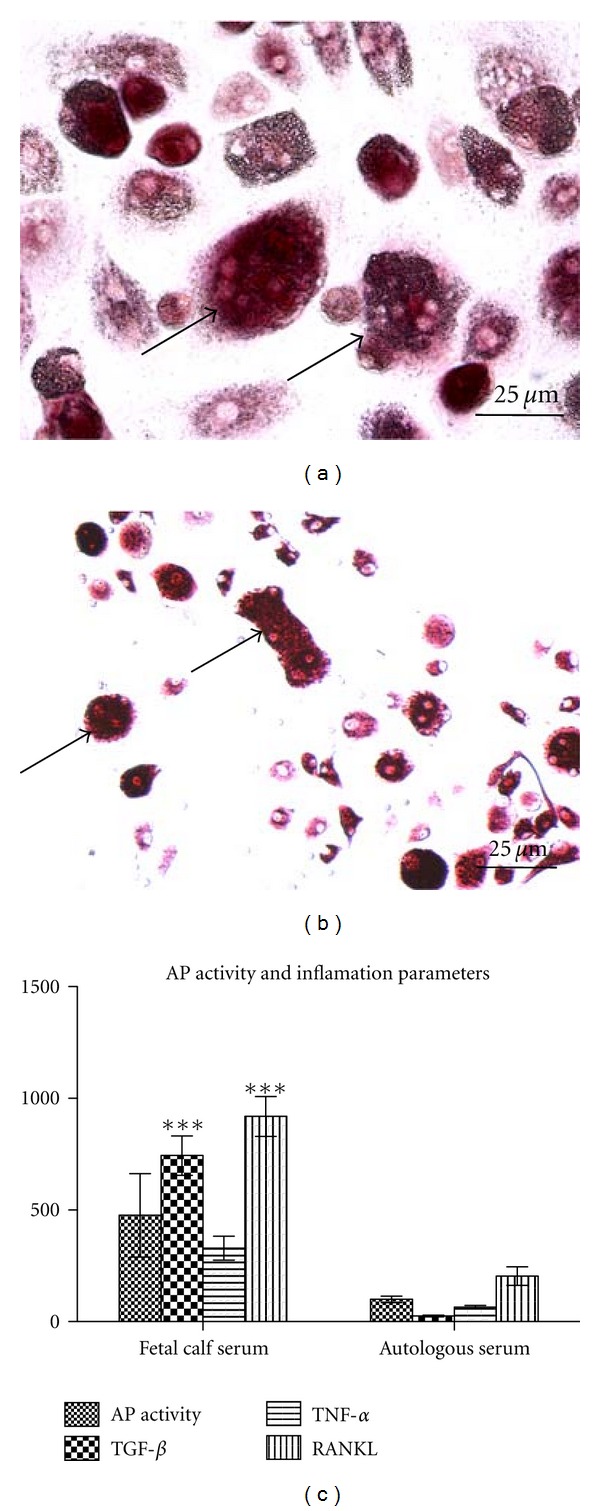
Differentiation procedure activates and maturates monocytes. TRAP staining of giant cells (marked with →) occurring after differentiation treatment of PCMOs with medium containing vitamin D (a) and osteoclasts (b); magnification 200x. (c) Comparison of Alkaline phosphatase [*μ*mol/min]-, TNF-alpha [pg/mL]-, TGF-beta [pg/mL], and RANKL-[pg/mL] levels normalized to SRB in PCMOs either isolated and differentiated in medium containing fetal calf or autologous serum (*N* = 5, *n* = 3). Differentiation performed with osteogenic medium containing vitamin D (*P < 0,05; ***P < 0.001).

To investigate if the observed giant cells represent foreign body giant cells or osteoclasts, we performed TRAP staining. Here, the strongly positive staining clearly identified these cells as osteoclasts (Figure 5(a); osteoclasts marked with →).

### 3.4. Activation of Monocytes Promotes Alkaline Phosphatase Activity

To investigate if the observed increase of AP activity in monocytes, MOMPs, and PCMOs during the differentiation procedure is a sign of monocyte activation and maturation into macrophages or osteoclasts, rather than of osteogenic transdifferentiation, we examined the activity of alkaline phosphatase in macrophages and osteoclasts. Here, alkaline phosphatase activity in macrophages showed similar levels to monocytes, MOMPs, and PCMOs after the osteogenic differentiation procedure. In contrast, maturation into osteoclasts did not substantially alter AP activity (Figure 3(a)).

To confirm the positive correlation between monocyte activation and increase in AP activity, we activated PCMOs—which reach the highest AP levels after differentiation treatment—by treatment with fetal calf serum (FCS). Here, the cultivation in presence of fetal calf serum leads to higher levels of proinflammatory cytokines TNF-alpha, TGF-beta, and RANKL in the cultures in comparison to cultivation in autologous serum. This indicates higher activation of the cells. Thereby, higher cell activation was accompanied with distinctly higher activity of alkaline phosphatase (Figure 5(c)).

## 4. Discussion

Differentiation of monocytes into osteoblasts would represent a transdifferentiation process, which is defined as conversion of a differentiated cell type into another type of fully differentiated cells. Transdifferentiation into bone tissue is a naturally occurring phenomenon, reported as metaplasia. Ectopic bone formation quite frequently occurs in scar tissue, in muscle after repeated trauma, and in walls of sclerotic arteries [15]. Usually, the transdifferentiation process includes a dedifferentiation and a redifferentiation step [16]. In case of monocytes, several treatment options are reported to shift these cells into a stem cell-like cell type, able to differentiate into various cell types [7, 9, 17, 18]. Investigation of suitability of monocytes as sources for generation of osteoblasts was performed on MOMPs and PCMOs, two reported cell types obtained from monocytes that can transdifferentiate into mesenchymal cell types. Both cell types are described as cells with a unique phenotype. While MOMPs are characterized by combined expression of CD14, CD45, CD105, CD34, and type I collagen, PCMOs show a combined expression of CD14, CD45, low levels of CD34, and CD90 [4, 10]. However, our study revealed that MOMPs additionally expressed CD90, while PCMOs were positive for CD105. Therewith both cell types were indistinguishable regarding the investigated markers and did not even differ substantially from untreated control monocytes after 6 days of culture.

The dedifferentiation process from monocytes towards PCMOs was characterized in more detail. Ungefroren et al. confirmed a change of the monocytes phenotype towards a more stem-cell-like appearance during a 6-day dedifferentiation treatment. The cells downregulated some monocyte-defining gens and started to express stem cell markers over time [19]. Recently, our group demonstrated that dedifferentiation of monocytes towards PCMOs with subsequent hepatogenic differentiation can be significantly improved by the use of the patient's autologous serum instead of human AB serum or fetal calf serum (FCS) [3]. However, within the present study, we could neither detect a positive nor a negative impact on susceptibility of PCMOs towards osteogenic differentiation by using autologous serum instead of either FCS or human AB serum during dedifferentiation treatment and/or subsequent osteogenic differentiation (data not shown). Moreover, our data suggest that although cells were treated towards MOMPs they undergo spontaneous partial dedifferentiation during a 6 day culture period and are finally comparable to PCMOs. Both cell types show downregulation of CD14 and upregulation of the stem cell markers CD34, CD90, and CD105 compared to freshly isolated monocytes [7, 9]. It is noteworthy that even untreated control monocytes undergo partial dedifferentiation during *in vitro* culture. This raises the question if dedifferentiation of PCMOs and MOMPs is dependent on a specific treatment only or rather the response to *in vitro* culture conditions [20].

The general accepted hypothesis of the molecular mechanism of transdifferentiation is the change in activity of a master switch gene, whose normal function is to distinguish both tissues in development [16]. In case of bone, Runx2 is associated as a master regulatory gene known to control most bone specific gene expression [21]. To fulfill criteria of transdifferentiation, this switch of master gene activity is paralleled by phenotypical cell changes with the loss of its original markers and the adoption of the markers of the new cell phenotype [15]. When we investigated the response of the monocyte-derived cells to osteogenic differentiation, the osteogenic medium failed to induce expression of osteogenic transcription factors and bone-related marker genes. Furthermore, it failed to induce production of detectable amounts of mineralized matrix. Instead of a phenotypic cell change towards an osteoblast phenotype, the hematopoietic surface markers CD14, CD45 and CD68 persisted during this culture process.

Noteworthy, our study revealed that expression of osteonectin, osteopontin, and osteocalcin—which was used in several studies to confirm osteogenic transdifferentiation of monocytes—is already expressed in freshly isolated monocytes [9, 17]. It is well known that osteopontin and osteonectin are expressed in various tissues [22, 23]. Furthermore, osteocalcin, which is known to be one of the most specific bone markers, is expressed in adipose and vascular tissues as well as in differentiated monocytes [24–26]. These findings underline that the expression of some apparently typical, but not tissue-specific markers does not necessarily imply the differentiation status of a cell. The differentiation of a cell is rather determined by unique expression pattern of a variety genes, which—considered separately—are not necessarily specific for one tissue [27]. Similarly, the appearance of alkaline phosphatase activity does not require transdifferentiation into osteoblasts, as this enzyme occurs in various tissues apart from bone, including myeloid cells such as neutrophil granulocytes and dendritic cells [28]. It is known that monocyte-derived granuloma cells are positive for bone-specific alkaline phosphatase *in vivo* and that alkaline phosphatase is expressed during monocyte differentiation *in vitro* [26, 29]. Our present work confirms that maturation of monocytes into macrophages is accompanied by an increased expression and activity of alkaline phosphatase. The spontaneous transformation of monocytes into osteoclasts *in vitro* is a well-known phenomenon [30, 31] as well as its alkaline phosphatase expression [26]. Our data confirms expression of alkaline phosphatase in osteoclasts on RNA level, yet it does not lead to measurable alkaline phosphatase activities in the cells.

When MOMPs and PCMOs were treated with osteogenic medium [9, 17] —we observed a strong apoptotic effect in these cells. This, however, is most likely linked to the presence of dexamethasone, which is known to trigger apoptosis [32]. In contrast, osteogenic differentiation medium containing vitamin D as substitute for dexamethasone did not influence cell viability negatively [33]. However, both used media failed to induce a proper osteogenic transdifferentiation in monocytes. Of course, we cannot rule out that monocytes inhere a transdifferentiation capacity towards osteoblasts using a more potent osteogenic medium.

It is likely that both supplements—dexamethasone and vitamin D—have different effects on monocytes as on mesenchymal stem cells and osteoblasts. Both substances show beneficial effects in myeloproliferative disorders. While dexamethasone leads to apoptosis, vitamin D promotes maturation of undifferentiated monocytes [34]. Thereby, vitamin D seems to favor maturation of monocytes into osteoclasts [35]. This is in line with our findings, as we have detected more osteoclasts after vitamin D treatment rather than after dexamethasone incubation and a decrease of stem cell markers CD34 and CD90 after treatment with both substances.

Summarizing our findings, we must conclude that we could not confirm inherent potential for osteogenic transdifferentiation of monocytes in our experimental setting. It seems more likely that the observed change of the cells in response to treatment towards PCMOs and MOMPs and subsequent differentiation procedure represents usual events in monocyte biology of maturation towards macrophages and osteoclasts. Of course, we cannot rule out that cells from monocytic origin have a potential to transdifferentiate into other cell types—including osteoblasts—in response to specific stimuli.

## Figures and Tables

**Table 1:** Sequences of used primer pairs and gen bank accession number of target genes.

Gene	Gene bank accession number	Forward primer 5′→3′	Reverse primer 5′→3′	Annealing temperature	Amplicon size [bp]
GAPDH	NM_002046.3	GTC AGT GGT GGA CCT GAC CT	AGG GGT CTA CAT GGC AAC TG	58°C	419 bp
CD34	NM_001025109	AGAAAGGCTGGGCGAAGACCC	AGTGGGGAAGGGTTGGGCGT	56°C	311 bp
Collagen Type 1	NM_000088.3	CAGCCGCTTCACCTACAGC	TTTTGTATTCAATCACTGTCTTGCC	56°C	84 bp
Runx2	NM_001015051.3	TGCCTAGGCGCATTTCAGGTGC	GGTGGTGGTGCATGGCGGAA	58°C	359 bp
Osterix	NM_152860	CCCAGGCAACACTCCTACTC	GGCTGGATTAAGGGGAGCAAA	62°C	175 bp
Osteonectin	NM_003118	AGCACCCCATTGACGGGTA	GGTCACAGGTCTCGAAAAAGC	60°C	105 bp
Osteopontin	NM_000582	CTCCATTGACTCGAACGACTC	CGTCTGTAGCATCAGGGTACTG	60°C	257 bp
Osteocalcin	NM_199173.3	ATGAGAGCCCTCACACTCCTC	GCCGTAGAAGCGCCGATAGGC	62°C	294 bp
Bone sialoprotein	NM_004967	TGACTCATCCGAAGAAAATGGAG	CTGGATTGCAGCTAACCCTGT	60°C	202 bp
Alkaline phosphatase	NM_000478.4	ACGTGGCTAAGAATGTCATC	CTGGTAGGCGATGTCCTTA	53°C	475 bp
Matrix Gla Protein	NM_000900	AGATGGAGAGCTAAAGTCCAAGA	GTAGCGTTCGCAAAGTCTGTA	60°C	102 bp
Osteoblast specific factor 2	NM_006475	TAAGTTTGTTCGTGGTAGCACC	GTGTGGGTCCTTCAGTTTTGATA	60°C	140 kb
BMP2	NM_001200	CCCCCTACATGCTAGACCTGT	CACTCGTTTCTGGTAGTTCTTCC	60°C	150 kb
BMP4	NM_130851	TGGTCTTGAGTATCCTGAGCG	GCTGAGGTTAAAGAGGAAACGA	60°C	130 kb
RANKL	NM_033012.3	TCCCAAGTTCTCATACCCTGA	CATCCAGGAAATACATAACACTCC	56°C	245 bp
Osteoprote-grin	NM_002546.3	CCGGAAACAGTGAATCAACTC	AGGTTAGCATGTCCAATGTG	60°C	313 bp

**Table 2:** Phenotype of monocyte-derived cells. Numbers indicating percentage of positive cells ± standard error of the mean (SEM) measured by FACS analysis from 3 independent donors.

	Monocytes 6 days	MOMPs 6 days	PCMOs 6 days	Monocytes 20 days	MOMPs 20 days	PCMOs 20 days	Osteo-blasts
CD14	11,16 ± 0,77	16,37 ± 8,62	33,62 ± 5,41	9,97 ± 1,83	19,69 ± 2,49	22,92 ± 7,69	3,52 ± 3,04
CD45	93,71 ± 4,29	85,72 ± 7,52	98,60 ± 0,45	85,59 ± 10,97	91,77 ± 6,29	97,49 ± 0,72	0,61 ± 0,36
CD90	19,83 ± 13,43	35,53 ± 17,76	32,24 ± 16,13	26,80 ± 21,02	16,98 ± 13,70	9,51 ± 4,38	96,35 ± 1,76
CD105	82,87 ± 10,56	86,29 ± 4,83	96,38 ± 0,91	69,44 ± 5,73	55,67 ± 13,12	61,85 ± 8,28	94,77 ± 1,78
